# Comparative analysis of gut microbiota and metabolome in captive Chinese and Malayan pangolins

**DOI:** 10.3389/fmicb.2025.1599588

**Published:** 2025-06-18

**Authors:** Chengwei Xiang, Luna Su, Mingzheng Han, Jieren Liang, Fanghui Hou, Jianzhao Liao

**Affiliations:** ^1^College of Veterinary Medicine, South China Agricultural University, Guangzhou, China; ^2^Guangdong Wildlife Monitoring and Rescue Center, Guangzhou, China

**Keywords:** Chinese pangolins, Malayan pangolins, gut microbiota, metabolomics Chinese pangolins, metabolomics, Wildlife

## Abstract

As an endangered species in the world, pangolins have attracted much attention due to their unique ecological value. In captivity, pangolins face numerous survival challenges, especially intestinal health problems, which are closely related to the gut microbiome and metabolome. The aim of this study was to contrast the gut microbiota and metabolome of Chinese pangolin and Malayan pangolin in captivity, which in order to explore the differences in digestive physiology and metabolic function between the two species. Through 16S rRNA gene sequencing and non-targeted metabolomics analysis, we identified significant differences in the composition and diversity of the gut microbiota between these two species. The gut microbiota of Chinese and Malayan pangolins is dominated by *Firmicutes* and *Proteobacteria* at phylum level. At the genus level, the abundance of *Clostridium sensu stricto 13* in Chinese pangolins was significantly higher than Malayan pangolins, whereas *Peptostreptococcus*, and *Blautia* are more abundant in Malay pangolins. Moreover, integrative analysis of the microbiome and metabolome revealed important correlations: *Peptostreptococcus* was positively correlated with 13-HpOTrE (r) metabolism, while *Clostridium sensu stricto 13* was negatively correlated with 13-HpOTrE (r) metabolism. *Blautia* was positively correlated with 1-phenylethylamine metabolism. These results provided important gut microbiome and metabolomics data for the conservation and artificial breeding of pangolins, which can help optimize captive pangolins feeding management and health maintenance.

## 1 Introduction

The pangolin is a unique and ancient mammal, belonging to the pangolin family of the order Squamata (Sun et al., 2024). The lifespan of pangolins typically ranges from 10 to 20 years, but in the wild they tend to live much shorter lives due to hunting and habitat loss (Gu et al., 2024). All species of pangolins face serious threats to their survival owing to illegal hunting and habitat destruction, and they are listed as endangered by the International Union for Conservation of Nature (IUCN) (Wang et al., 2022). The Chinese pangolin inhabits the ground, dig holes, and occasionally climb trees. It can clearly locate termite nests up to two meters deep underground through its keen sense of smell. The Malay pangolin prefer to live in trees, have a slender figure, and are adapted to living in trees. These differences help to distinguish the two species and understand their biology and ability to adapt to their environment (Lan et al., 2025). Currently, both Chinese and Malayan pangolin stocks in China are very rare and both are rapidly declining. Therefore, a number of works related to the protection of captive pangolins has been carried out in China, with a view to playing a positive role in scientific research and breeding, promoting the recovery of wild populations, and protecting biodiversity and ecological balance (Hua et al., 2015).

The gut microbiota of pangolins fulfills a fundamental role in their overall health, especially in captivity. In the wild, pangolins have evolved to have a specific gut microbiota that is closely associated with their natural diet, which mainly consists of ants and termites (Jiao et al., 2022). This natural gut microbiota plays an important role in the digestion of these insects, which are rich in chitin and other complex substances. In addition, a diverse and stable gut microbiota can outcompete harmful bacteria, fungi, and viruses for resources and attachment sites in the gut (Moutsoglou et al., 2025). Moreover, gut health is closely related to the reproductive performance (Lin et al., 2025). However, captive pangolins are often fed diets that may not be exactly the same as their natural diet in the wild. For instance, the diet in captivity contains more processed foods or different types of insects, and the intestinal bacterial structure and digestion of related nutrients have changed compared with that in the wild, which remains to be investigated for the growth and development of pangolins in captivity and the maintenance of their physical condition (Hua et al., 2015). Furthermore, the gut microbiota acts as a crucial part of the immune system in pangolins (Dai et al., 2024). Many captive pangolins are in contact with human caretakers, and there is a risk of cross-infection (Hou et al., 2023). Therefore, understanding and maintaining the gut health of captive pangolins is critical to their survival, growth, reproduction and overall health, which directly affects the success of conservation efforts in captive breeding and recovery programs for pangolins.

To sum up, the gut health of pangolins is closely relevant to the composition and function of their gut microbes, and the effects of captive and wild environments on gut microbes are special factors to be considered when protecting pangolins. The overall target of this study is to comprehensively explore the gut health of captive Chinese and Malayan pangolins using 16S rRNA sequencing and metabolomics techniques, which provide scientific proof for the amelioration of their captive management and conservation.

## 2 Materials and methods

### 2.1 Animal population

This research encompassed pangolins rescued from various counties in Guangdong Province. From 2021 to 2024, these pangolins were brought to the Guangdong Provincial Wildlife Rescue Monitoring Center by local government officials and the public. Every single animal was placed in isolation and quarantine for a minimum of 1 month. In cases where the pangolins had injuries or other medical issues, they received treatment until they seemingly regained good health. A pangolin was regarded as being in good health when it was active, had a healthy appetite, excreted normal feces, was free from parasites and traumatic injuries, and maintained a stable weight.

In the adult Chinese pangolins (CP), 14 males and 14 females were identified. The body mass of these individuals exhibited a range from 3.1 kg to 5.96 kg. For the adult Malay pangolin (MP), it was composed of 13 males and 13 females, with their weights fluctuating within the interval of 2.94 kg to 8.87 kg. For each species, six pangolins with similar body lengths and close body weights were selected, with an equal number of males and females (three male and three female Chinese pangolins; three male and three female Sunda pangolins). The fresh feces were collected for subsequent experimental analysis.

### 2.2 Microbial 16S RNA gene sequencing

Fecal samples were collected from Chinese and Malay pangolins. Genomic DNA isolation was performed using a specialized Fecal Genome DNA Extraction kit (AU 46111-96, Bio Teke, China), followed by electrophoretic quality assessment total DNA was amplified by PCR using universal primers (F: 5′-CCTACGGGNGGCWGCAG-3′; R: 5′-GACTACHVGGG TATCTAATCC-3′). The PCR product was purified and quantified for up-sequencing. The raw data were filtered, and sequencing primers were removed from the de-multiplexed sequences using cutadapt (v1.9). The remaining reads were then merged using FLASH. Chimeric sequences were filtered using with Vsearch software (v2.3.4). Finally, denoising processing with DADA2 to generate amplicon sequence variants (ASVs). Alpha and beta diversity analyses were conducted based on the obtained ASV feature sequences and ASV abundance tables. The species abundance in each stratum of each sample was then quantified according to the ASV abundance scale, comparative intergroup analysis of microbial compositional disparities using nonparametric statistical methods.

### 2.3 Fecal metabolome analysis

Precisely weighed fecal aliquots (25 ± 1 mg) underwent standardized metabolite extraction procedures. Initial homogenization was performed with 500 mL of extraction solution [MEOH: CAN: H_2_O, 2:2:1, (v/v)] containing beads, utilizing three cycles of mechanical agitation (30 s vortexing) interspersed with 5-min ultrasonic treatments in a refrigerated bath (4°C), and the steps were repeated three times. Following phase separation, samples underwent cryoprecipitation at −40°C. Phase-separated mixtures were subjected to high-speed centrifugation [12,000 rpm, RCF = 13,800 (g), *R* = 8.6 cm] for 15 min at 4°C. The resulting supernatant was carefully transferred to pre-cleaned glass vials using positive displacement. Quality control (QC) samples were generated through combinatorial pooling of equivalent supernatant volumes from all experimental groups.

LC-MS/MS analyses were performed using an UHPLC system (Vanquish, Thermo Fisher Scientific) with a Waters ACQUITY UPLC BEH Amide (2.1 mm × 50 mm, 1.7 μm) coupled to Orbitrap Exploris 120 mass spectrometer (Orbitrap MS, Thermo). After the raw data were converted into mzXML via ProteoWizard software, followed by comprehensive feature processing through a customized R-based pipeline integrating XCMS algorithms. This workflow executed peak detection, feature extraction, chromatogram alignment, and signal integration through optimized parameter settings. And then matched with the self-constructed secondary mass spectrometry database for the annotation of the substances, and the cutoff value of the algorithmic scoring was set at 0.3.

Statistical analysis was executed in R (version 4.0.0). The raw protein intensities were normalized using the “medium” method. Hierarchical clustering was conducted with the pheatmap package, while principal component analysis (PCA) was carried out with the metaX package. PLS-DA analysis was performed with the ropls package, and generated variable importance projection (VIP) indices. Intervariable associations were assessed through Pearson correlation matrices from the cor package. The final metabolite with significant differences were selected based on employing a tripartite criteria: *p*-value <0.05, fold change >1.2 (obtained by *t*-test), and VIP values calculated through PLS-DA. Functional annotation for the protein sequences utilized hypergeometric-based enrichment analysis with KEGG pathway.

### 2.4 Correlation analysis of microbes and metabolome

In order to investigate the phenotypic alterations potentially induced by shifts in the structure of the host microbial community, correlation analyses between the metabolome and microbiome were carried out. These analyses involved Pearson’s correlation analysis, correlation network diagram analysis, and correlation Sankey diagram analysis. To further identify the hub microbial taxa, we visualized the association network between microbial taxa and metabolites using Cytoscape 3.10.1, and subsequently analyzed the network with the CentiScaPe, Analyze Network, and MCODE plugins for screening. A significance level of *p* < 0.05 was regarded as statistically significant.

## 3 Results

### 3.1 Microbial diversity analysis of Chinese and Malay pangolins

**Figure 1:**
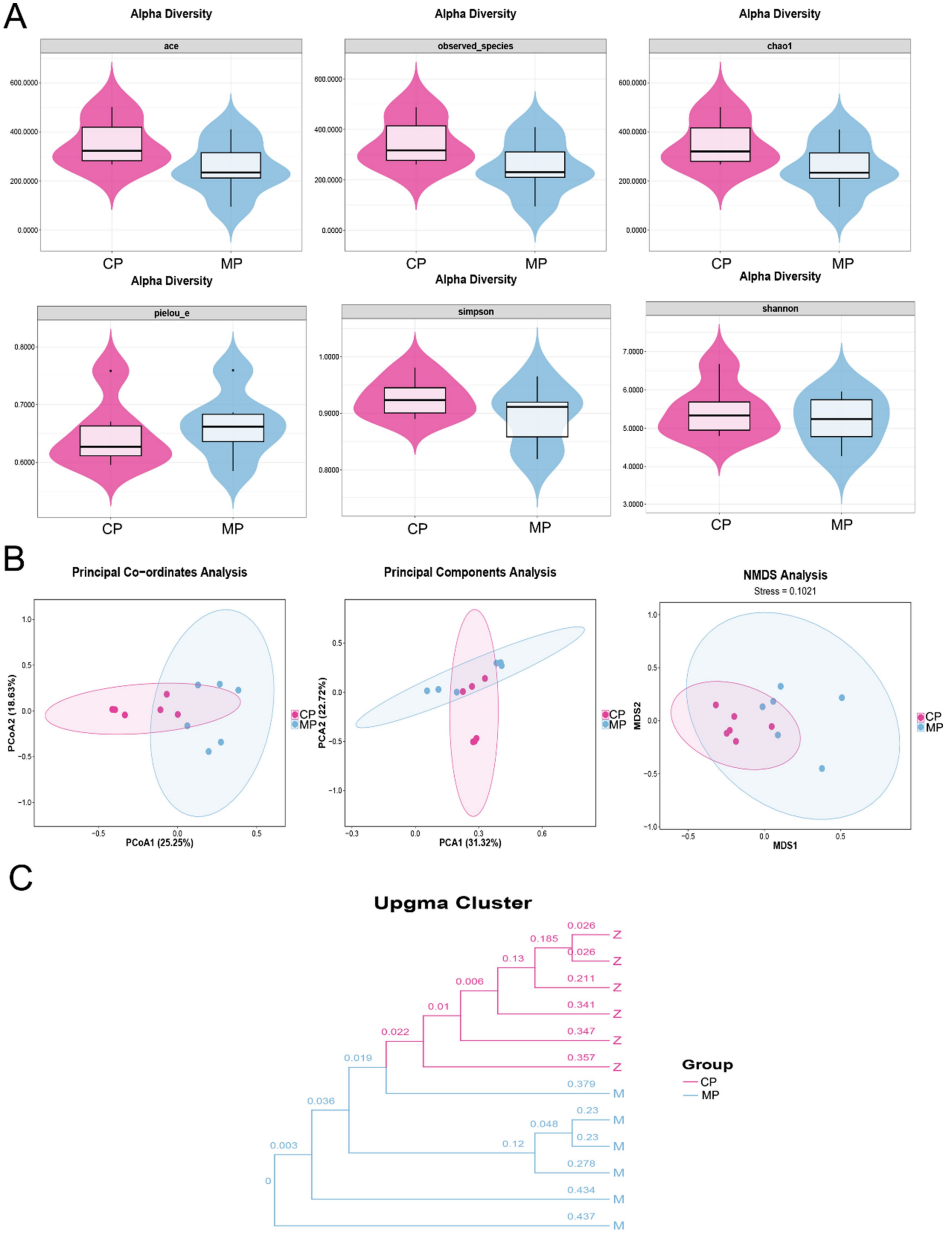
The diversity analysis of intestinal flora in Chinese and Malay pangolins. **(A)** Alpha diversity analysis (ACE, observed_species, Chao1, pielou-e, Simpson, and Shannon). **(B)** Beta diversity analysis (PCoA, PCA, and NMDS). **(C)** Bacterial cluster analysis.

The analysis of gut microbiota diversity was shown in Figure 1. Here, the average value of ACE, observed_species, Chao1, Simpson, and Shannon of gut microbiota in Chinese pangolins were higher than that of Malay pangolins, and the average value of pielou-e in Chinese pangolins was lower than that in Malay pangolins. However, there was no significant difference between the species richness and multiplicity of microorganisms in the intestinal contents of the two species based on the Alpha diversity analysis (Figure 1A). Additionally, to assess beta diversity, we used PCA, PCoA, and NMDS plots. As shown in Figure 1B, the plots in CP group were not independent of the MP group, which suggested that the structure of gut microflora was no significant difference in the flora structure. In addition, the similitude among the samples was shown in the modality of hierarchical tree through the cluster analysis, and the clustering effect is measured by the sub-division length of the cluster tree. We found that the flora samples of the two species of pangolin were concentrated separately (Figure 1C).

### 3.2 Microflora structure differential analysis of Chinese and Malay pangolins based on phylum level

**Figure 2:**
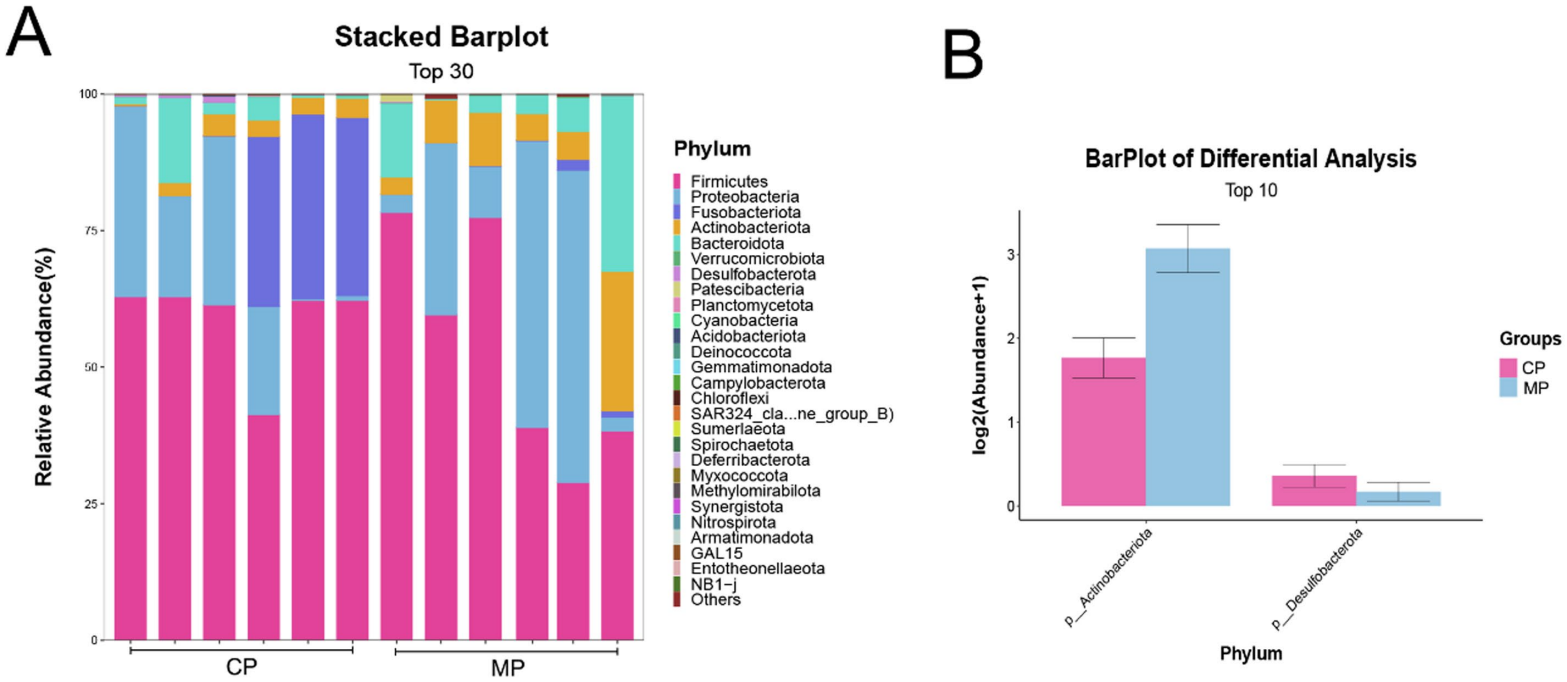
The intestinal flora at phylum level in Chinese and Malay pangolins. **(A)** The stacked barplot of gut microbiota at phylum level. **(B)** The differential analysis of gut microbiota at phylum level.

As shown in Figure 2A, we found that there was without significant difference between the flora structure of the Chinese and Malay pangolins at the phylum aspect through the analysis of the flora of the two groups, and *Firmicutes* and *Proteobacteria* were accounted for a high proportion. Although the flora structure is similar, there are still some differences in the microbiota content between the Chinese and Malay pangolins. As presented in Figure 2B, the *Actinobacteriota* was prominently increased and the *Desulfobacterota* was prominently decreased in MP group compared to CP group.

### 3.3 Microflora structure differential analysis of Chinese and Malay pangolins based on genus level

**Figure 3:**
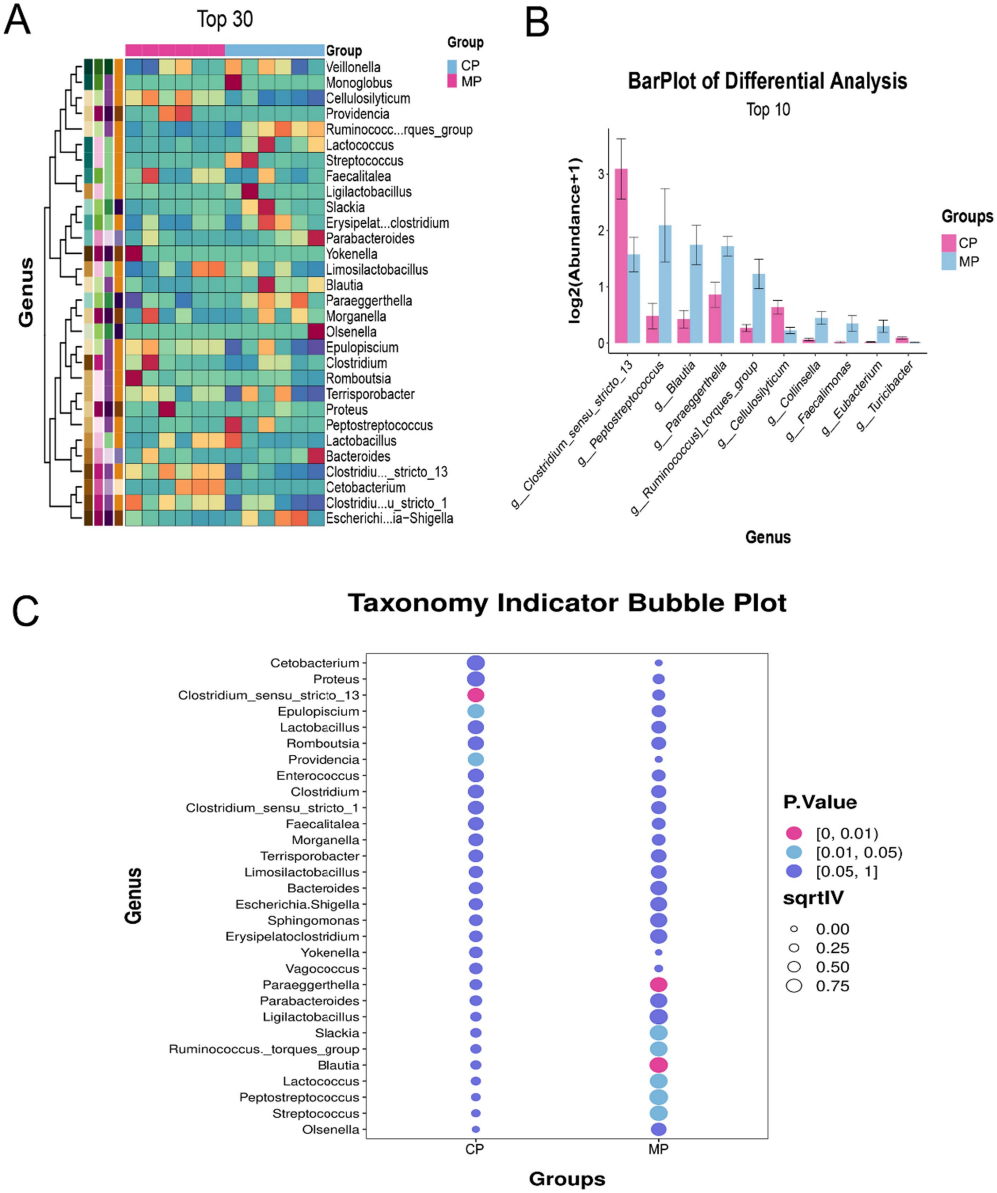
The differential analysis of gut microbiota at genus level. **(A)** Heatmap analysis of differential gut microbiota at genus level. **(B)** The barplot of differential gut microbiota at genus level. **(C)** The indicator species analysis of differential gut microbiota at genus level.

The categorization and analysis of the microflora at genus were displayed in Figure 3. Here, we showed the top 30 different bacteria genera through the heatmap (Figure 3A), and the top 10 different bacteria genera through the bar chart (Figure 3B), which found that the *Clostridium sensu stricto 13*, *Peptostreptococcus*, and *Blautia* were the microbiota with the most significant changes at genus level. Among them, *Clostridium sensu stricto 13* in CP group was obviously more than that in MP group, at the same time that *Peptostreptococcus*, and *Blautia* in CP group were significantly reduced (Figure 3C).

### 3.4 Metabolite analysis of Chinese and Malay pangolins intestinal flora based on metabonomics

**Figure 4:**
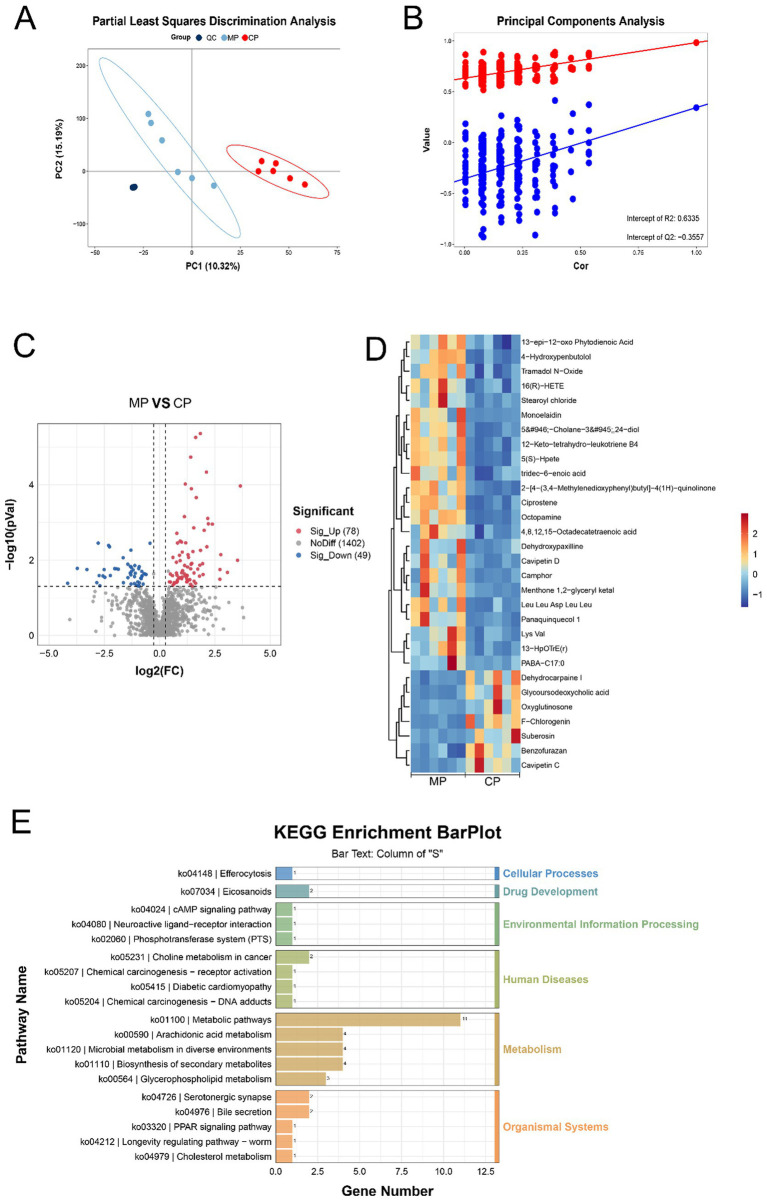
The differential analysis of flora metabolites. **(A)** PLS-DA analysis. **(B)** Permutation test diagram. **(C)** Volcano graph of the differential metabolites. **(D)** Heatmap of the differential metabolites. **(E)** Pathways of metabolite enrichment with KEGG analysis.

Subsequently, the metabolomics technique was used to analyze the types of metabolites in the flora and predicted the biological functions of the differential metabolites. Pattern discriminant analysis was performed through PLSDA and PCA. The clustering of QC plots in PLSDA indicates that the analytical method has high replayability and low alterability. Additionally, the plots standing for the MP group were signally disunited from those of the CP group, which interpreted a significant variation in the metabolites and modeling success (Figures 4A,B). Compared with Chinese pangolins, there were 78 significantly up-adjusted and 49 down-adjusted metabolites in Malay pangolins (Figure 4C). Partial differential metabolites are displayed in the heat map (Figure 4D). KEGG analysis displayed that differential metabolites were participated in metabolic processes like endocytosis, arachidonic acid metabolism and bile secretion (Figure 4E).

### 3.5 Analysis of the association between metabolomics and intestinal differential flora

**Figure 5:**
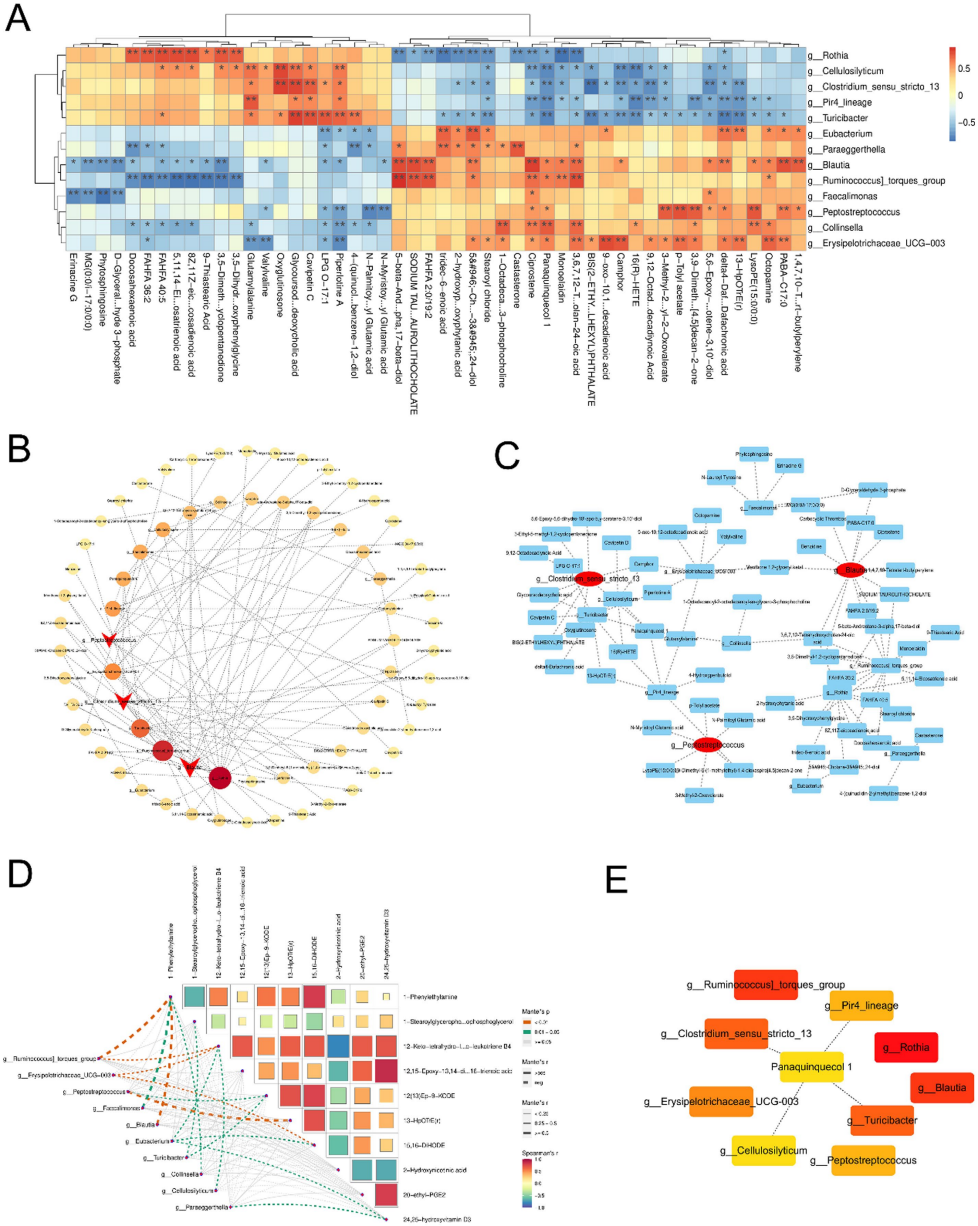
Correlation analysis of different flora and metabolites. **(A)** Heatmap showed the significant correlation between bacterial flora and metabolites. **(B,C)** The bubble map showed the significant correlation between pivotal bacteria and metabolites. **(D)** Mantel test for pivotal bacteria and metabolites. **(E)** Associations between the key metabolites and flora.

Based on 16S rRNA sequencing and metabolomics, we performed a comprehensive joint analysis of differentially abundant bacterial genera and metabolites. Correlation heatmaps were utilized to visualize the relationships between these taxa and metabolites (Figure 5A). To further identify key microbial hubs, we employed advanced network analysis tools such as CentiScaPe, Analyze Network, and CytoHubba within Cytoscape for enhanced visualization and screening. Our results declared that, contrasted with the MP group, *Clostridium sensu stricto 13*, *Peptostreptococcus*, and *Blautia* exhibited significant differences as hub genera. The Mantel test was used to evaluate the relativity between differential genera and metabolites. Specifically, *Peptostreptococcus* was positively correlated with 13-HpOTrE (r) metabolism, while *Clostridium sensu stricto 13* was negatively correlated with 13-HpOTrE (r) metabolism. *Blautia* was positively correlated with 1-phenylethylamine metabolism (Figures 5B–E).

## 4 Discussion

Due to extensive poaching and globally illegal transaction, pangolins are one of the most endangered animals. Two species of Asian pangolins, the Malayan pangolin and the Chinese pangolin, are mainly distributed in Southeast Asia and are the most endangered and most at risk of extinction (Nie et al., 2009). Therefore, captive breeding has been utilized as an efficacious method to protect endangered pangolins. Nevertheless, keeping pangolins in captivity poses huge challenges. By reason of the captive environment and food supply are different from the wild habitat, gastrointestinal diseases have become the leading cause of death in captive pangolins (Chang et al., 2021). The gut microbiome can affect the nutritional metabolism and immune response of the host, and fulfill an important role in the physiological and pathological state of the host (Marquez-Paradas et al., 2025). Previous researches have shown that discrepancies in the gut microbiome among wild and captive pangolins can affect holistic health. Meanwhile, it has found that there are some differences in the nutritional metabolic capacity of intestinal flora, virulence factors and the abundance of antibiotic resistance genes between Chinese and Malay pangolins under artificial domestication (Dai et al., 2024; Jiao et al., 2022). However, the structural differences are unclear. Therefore, through the analysis of flora structure and metabolomics, the key bacteria and metabolites affecting the intestinal health of Chinese and Malay pangolins in captivity were investigated.

In this study, we analyzed the species richness, diversity and differences of the intestinal flora of Chinese and Malay pangolins via the alpha and beta diversity analysis. Here, we found that the value of ACE, observed species, and Chao1 in gut microbiota were increase in Chinese pangolins compared to Malay pangolins. Chao1, observed species and ACE indices mainly reflect community richness, so the species richness of the gut flora of the Chinese pangolin is more than of which the Malay pangolin. In addition, Shannon and Simpson mainly reflect community diversity. The greater the value, the greater the uncertainty, which means the more unknown factors in the community, that is, the higher the diversity (Li et al., 2022). Thus, the relatively high Shannon and Simpson indexes in Chinese pangolin also indicated that the abundance of intestinal flora of Chinese pangolin was higher than that of Malay pangolin. Furthermore, the pielou-e index also reflected that the community evenness of gut microbiota in Chinese pangolin was lower than that in Malay pangolin. Although beta analysis showed no significant differences between the groups, the cluster tree could visually show that the intestinal flora samples between the two groups were highly similar within the group. Thus, although there was no significant difference between intestinal flora richness and multiplicity of the two varieties of pangolin after captivity, they still had variety specificity.

It has been reported that the differential gut flora has potential implications for the health of the animals. At phylum level, the *Actinobacteriota* was significantly increased and the *Desulfobacterota* was significantly decreased in MP group contrasted to CP group. Additionally, the *Clostridium sensu stricto 13*, *Peptostreptococcus*, and *Blautia* were the microflora with the most outstanding variations at the genus level.

*Clostridium sensu stricto 13* is an indispensable component of the intestinal microbiota, playing a crucial role in maintaining gut flora homeostasis and regulating animal metabolism (Liu et al., 2025). Studies have shown that species of *Clostridium* are indispensable for maintaining the intestinal of the barrier integrity and synthesizing short-chain fatty acids (SCFAs) like acetate and butyrate, which significantly affect the immune system and metabolic processes in animals (Adjele et al., 2024). A recent investigation into the effects of protein restriction on weanling piglets showed that both protein restriction and subsequent supplementation could cause morphological changes, alterations in microbial composition, and charges in metabolite profiles in the small intestine. *Clostridium sensu stricto 13* has become one of the key species linked to these intestinal microbiota modifications (Shi et al., 2019). Therefore, *Clostridium sensu stricto 13* is able to play a crucial role in sustaining the equilibrium of intestinal microecology. It has the potential to inhibit the overgrowth of pathogenic flora, thereby promoting gut health. Moreover, it may enhance the overall health of pangolins by modulating gut immune responses, particularly by modulating the inflammatory processes of Chinese pangolin.

*Peptostreptococcus*, an anaerobic bacterium, which commonly found in the gastrointestinal tracts of animals. Research has demonstrated that commensal *Peptostreptococcus* species production of the tryptophan metabolite indoleacrylic acid (IA) that promotes intestinal epithelial barrier function and mitigate inflammatory responses (Wlodarska et al., 2017). These bacteria contribute to maintaining gut microbiota balance. Specifically, *Peptostreptococcus* is closely related to body weight regulation, glucose homeostasis, and the functionality of the intestinal immune system (Tian et al., 2022). Therefore, the proliferation of *Peptostreptococcus* in the intestinal tract may regulate the intestinal immune response, reduce the production of inflammatory mediators, and enhance intestinal barrier function, thus improving the disease resistance of Malayan pangolins.

*Blautia* is a significant genus within the gut microbiota, playing a critical role in sustaining gut health and metabolic processes. The metabolites produced by *Blautia*, especially SCFAs, are believed to exert important regulatory effects on energy metabolism, immune modulation, and inflammatory responses in animals (Zhao et al., 2025). A study showed that herbal preparations targeting specific gut microbiota could alleviate the metabolic problems of obese and diabetes rats by regulating gut microbiota, highlighting the indispensable role of *Blautia* in this process (Tian et al., 2022). Therefore, *Blautia* likely plays a focal role in the immune system of pangolins by modulating immune responses and alleviating intestinal inflammation, thereby promoting overall health. Moreover, the metabolites produced by *Blautia* can maintain the balance of the intestinal microecology, preventing the overgrowth of pathogenic microorganisms and ensuring the overall health of Malayan pangolins.

Furthermore, the integration of 16S rRNA sequencing and metabolomics analysis showed that *Clostridium sensu stricto 13* and *Peptostreptococcus* is closely related to 13-HpOTrE(r) metabolism, while *Blautia* is linked to 1-phenylethylamine metabolism. At present, the role of 13-HpOTrE (r) in the gut has not been reported. However, some researchers have found that 13-HpOTrE (r) has an excellent moderation effect on cytotoxicity and apoptosis. 13-HpOTrE (r) diminished the release of interleukin-6 and decreased the viability of cancer cells (Wolff et al., 2019). In the field of maintaining intestinal barrier function, 13-HpOTrE (r) may enhance the expression and assembly of tight junction proteins (such as occludin, claudin, etc.) between intestinal epithelial cells by activating specific intracellular signaling pathways, thereby reducing intestinal permeability for sustaining the integrity of the wholeness barrier in an all-round way. Effectively block intestinal bacteria, toxins and other harmful substances into the blood circulation, to prevent the occurrence of inflammation. In addition, 1-phenylethylamine, as a trace amine, can act on intestinal smooth muscle and stimulate intestinal contraction (Murata et al., 2009). It has been found to have contractile effects on the ileum of guinea pigs and rats, and this effect is independent of norepinephrine, acetylcholine, histamine and serotonin receptors. At the same time, 1-phenylethylamine can relax the mesenteric blood vessels, thereby improving blood flow in the intestine. When the body ingests foods containing 1-phenylethylamine, it may help in the digestive process by restricting blood flow to other parts of the body, diverting blood to the intestines. Thus, the abundant *Clostridium sensu stricto 13* in the intestine of the Chinese pangolin may interfere with the metabolism of 13-HpOTrE (r), affecting the intestinal barrier function. This might necessitate compensation for the barrier function by other microbiota- derived metabolites. In contrast, the dominant bacteria *Peptostreptococcus* and *Blautia* in the intestine of the Malayan pangolin can promote the metabolism of 13-HpOTrE (r) and 1-phenylethylamine.

## 5 Conclusion

Our study suggested that *Clostridium sensu stricto 13*, *Peptostreptococcus* and *Blautia* may influence gut health through their respective roles in the metabolic pathways of 13-HpOTrE (r) and 1-phenylethylamine between Chinese pangolin and Malayan pangolin. These provided important gut microbiome and metabolomics data for the conservation and artificial breeding of pangolins, which is helpful to optimize the feeding management and health maintenance of captive pangolins.

## Data Availability

The data presented in the study are deposited in the Figshare repository: https://doi.org/10.6084/m9.figshare.29245067.v1.

## References

[ref1] Adjele J. Devi P. Kumari P. Yadav A. Tchuenchieu K. A. Mouafo H. T.. (2024). Exploring the influence of age and diet on gut microbiota development in children during the first 5 years: a study from Yaounde, Cameroon. Front. Microbiol. 15:1512111. doi: 10.3389/fmicb.2024.1512111 39744404 PMC11688346

[ref2] Chang Y. C. Lin Z. Y. Lin Y. X. Lin K. H. Chan F. T. Hsiao S. T.. (2021). Canine parvovirus infections in Taiwanese pangolins (*Manis pentadactyla pentadactyla*). Vet. Pathol. 58, 743–750. doi: 10.1177/03009858211002198, PMID: 33866880

[ref3] Dai Z. Xie B. Xie C. Xiang J. Wang X. Li J.. (2024). Comparative metagenomic analysis of the gut microbiota of captive pangolins: a case study of two species. Animals 15:57. doi: 10.3390/ani15010057, PMID: 39795000 PMC11718824

[ref4] Gu T. Hu J. Yu L. (2024). Evolution and conservation genetics of pangolins. Integr. Zool. 19, 426–441. doi: 10.1111/1749-4877.12796, PMID: 38146613

[ref5] Hou Y. J. Chiba S. Leist S. R. Meganck R. M. Martinez D. R. Schafer A.. (2023). Host range, transmissibility and antigenicity of a pangolin coronavirus. Nat. Microbiol. 8, 1820–1833. doi: 10.1038/s41564-023-01476-x 37749254 PMC10522490

[ref6] Hua L. Gong S. Wang F. Li W. Ge Y. Li X.. (2015). Captive breeding of pangolins: current status, problems and future prospects. ZooKeys 507, 99–114. doi: 10.3897/zookeys.507.6970, PMID: 26155072 PMC4490220

[ref7] Jiao W. Liu L. Zeng Z. Li L. Chen J. (2022). Differences in gut microbes in captive pangolins and the effects of captive breeding. Front. Microbiol. 13:1053925. doi: 10.3389/fmicb.2022.1053925, PMID: 36560954 PMC9763570

[ref8] Lan T. Tian Y. Shi M. Liu B. Lin Y. Xia Y.. (2025). Enhancing inbreeding estimation and global conservation insights through chromosome-level assemblies of the Chinese and Malayan pangolin. GigaScience 14:giaf003. doi: 10.1093/gigascience/giaf003, PMID: 39947250 PMC11825179

[ref9] Li Z. Zhou J. Liang H. Ye L. Lan L. Lu F.. (2022). Differences in alpha diversity of gut microbiota in neurological diseases. Front. Neurosci. 16:879318. doi: 10.3389/fnins.2022.879318, PMID: 35837118 PMC9274120

[ref10] Lin X. Yu Z. Liu Y. Li C. Hu H. Hu J. C.. (2025). Gut-X axis. iMeta 4:e270. doi: 10.1002/imt2.270, PMID: 40027477 PMC11865426

[ref11] Liu S. Du J. Chen Y. Fan Q. Yue X. Zhao L.. (2025). Impact of gender and reproductive states on diets and intestinal microbiota in Pratt’s leaf-nosed bats (*Hipposideros pratti*). Comp. Biochem. Physiol. D 54:101459. doi: 10.1016/j.cbd.2025.101459, PMID: 40036980

[ref12] Marquez-Paradas E. Torrecillas-Lopez M. Barrera-Chamorro L. Del Rio-Vazquez J. L. Gonzalez-de la Rosa T. Montserrat-de la Paz S. (2025). Microbiota-derived extracellular vesicles: current knowledge, gaps, and challenges in precision nutrition. Front. Immunol. 16:1514726. doi: 10.3389/fimmu.2025.1514726 40051622 PMC11882860

[ref13] Moutsoglou D. Ramakrishnan P. Vaughn B. P. (2025). Microbiota transplant therapy in inflammatory bowel disease: advances and mechanistic insights. Gut Microbes 17:2477255. doi: 10.1080/19490976.2025.2477255, PMID: 40062406 PMC11901402

[ref14] Murata M. Katagiri N. Ishida K. Abe K. Ishikawa M. Utsunomiya I.. (2009). Effect of beta-phenylethylamine on extracellular concentrations of dopamine in the nucleus accumbens and prefrontal cortex. Brain Res. 1269, 40–46. doi: 10.1016/j.brainres.2009.03.002, PMID: 19285043

[ref15] Nie W. Wang J. Su W. Wang Y. Yang F. (2009). Chromosomal rearrangements underlying karyotype differences between Chinese pangolin (*Manis pentadactyla*) and Malayan pangolin (*Manis javanica*) revealed by chromosome painting. Chromosom. Res. 17, 321–329. doi: 10.1007/s10577-009-9027-0, PMID: 19283495

[ref16] Shi Q. Zhu Y. Wang J. Yang H. Wang J. Zhu W. (2019). Protein restriction and succedent realimentation affecting ileal morphology, ileal microbial composition and metabolites in weaned piglets. Animal 13, 2463–2472. doi: 10.1017/S1751731119000776, PMID: 31084646

[ref17] Sun S. Wei S. Dou H. Chen S. Gao H. Yang J.. (2024). Identifying habitat modification by Chinese pangolin in subtropical forests of southern China. Integr. Zool. 20, 361–375. doi: 10.1111/1749-4877.12862, PMID: 39040030 PMC11897934

[ref18] Tian P. Wu L. Kudo M. Hayashi M. Qin L. Gao M.. (2022). TangNaiKang, herbal formulation, alleviates obesity in diabetic SHR/cp rats through modulation of gut microbiota and related metabolic functions. Pharm. Biol. 60, 2002–2010. doi: 10.1080/13880209.2022.2096075, PMID: 36226871 PMC9578476

[ref19] Wang X. M. Janssens G. Xie C. G. Xie B. W. Xie Z. G. He H. J.. (2022). To save pangolins: a nutritional perspective. Animals 12:3137. doi: 10.3390/ani12223137, PMID: 36428365 PMC9686612

[ref20] Wlodarska M. Luo C. Kolde R. D'Hennezel E. Annand J. W. Heim C. E.. (2017). Indoleacrylic acid produced by commensal *Peptostreptococcus* species suppresses inflammation. Cell Host Microbe 22, 25–37. doi: 10.1016/j.chom.2017.06.007 28704649 PMC5672633

[ref21] Wolff C. Zoschke C. Kalangi S. K. Reddanna P. Schafer-Korting M. (2019). Tumor microenvironment determines drug efficacy *in vitro*—apoptotic and anti-inflammatory effects of 15-lipoxygenase metabolite, 13-HpOTrE. Eur. J. Pharm. Biopharm. 142, 1–7. doi: 10.1016/j.ejpb.2019.06.003, PMID: 31176725

[ref22] Zhao Z. Hu Z. Li L. (2025). Cardiac energy metabolic disorder and gut microbiota imbalance: a study on the therapeutic potential of Shenfu injection in rats with heart failure. Front. Microbiol. 16:1509548. doi: 10.3389/fmicb.2025.1509548, PMID: 40071211 PMC11895768

